# Gestational hypertension and risk of atopic diseases in offspring, a national-wide cohort study

**DOI:** 10.3389/fped.2023.1283782

**Published:** 2023-11-23

**Authors:** Chun-Ti Yang, Ching-Heng Lin, Ming-Chih Lin

**Affiliations:** ^1^ Children’s Medical Center, Taichung Veterans General Hospital, Taichung, Taiwan; ^2^ Department of Medical Research, Taichung Veterans General Hospital, Taichung, Taiwan; ^3^ Department of Post-Baccalaureate Medicine, College of Medicine, National Chung Hsing University, Taichung, Taiwan; ^4^ School of Medicine, National Yang Ming Chiao Tung University, Taipei, Taiwan; ^5^ Department of Food and Nutrition, Providence University, Taichung, Taiwan; ^6^ School of Medicine, Chung Shan Medical University, Taichung, Taiwan

**Keywords:** gestational hypertension, asthma, atopic dermatitis, allergic rhinitis, urticaria

## Abstract

**Introduction:**

Gestational hypertension can lead to complications, such as preeclampsia. Preeclampsia is one of leading causes of perinatal morbidity and mortality. Abnormal placentation, immune dysregulation, and maternal inflammatory response are possible etiologies. The burden of atopic diseases is increasing worldwide. Prenatal exposure might play a role in the pathogenesis of these two diseases. The aim of this study was to evaluate the association between gestational hypertension and atopic diseases from a nationwide perspective.

**Material and methods:**

The primary data were retrieved from Taiwan's National Health Insurance Research Database. The Maternal and Child Health Database was used to generate links between mothers and children. From 2004 to 2019, mothers with a diagnosis of gestational hypertension were identified as cases. The control groups were matched to the cases by maternal age, neonatal gender, date of birth, at a control-to-case ratio of 4:1. Each child was reviewed to confirm the diagnosis of atopic disease. Covariates including both maternal and neonatal factors were also collected.

**Results:**

A total of 1,935,874 primiparas were enrolled in this study. After excluding 16,851 mothers with a history of hypertension, a total of 1,919,023 offspring were included in the study for the period 2004–2019. Gestational hypertension was associated with asthma (HR, 1.12, 95% CI, 1.02–1.23) and atopic dermatitis (HR, 1.10, 95% CI, 1.00–1.21) in offspring after controlling for cofactors. Nevertheless, gestational hypertension did not play an independent factor for allergic rhinitis (HR, 1.02, 95% CI, 0.95–1.10) or urticaria (HR, 1.02, 95% CI, 0.91–1.15).

**Conclusion:**

Maternal gestational hypertension increases the cumulative risk for asthma and atopic dermatitis in offspring.

## Introductions

Gestational hypertension is defined by the American College of Obstetricians and Gynecologists as a systolic blood pressure greater than or equal to 140 mmHg or diastolic pressure greater or equal to 90 mmHg on two separate occasions at least four hours apart after 20 weeks of pregnancy when previous blood pressure was normal (1). If left untreated, gestational hypertension can lead to complications, such as preeclampsia, a serious condition that can cause organ damage and threaten the health of both mothers and babies. Currently, preeclampsia remains one of the leading causes of perinatal maternal and infantile morbidity and mortality (2). Although further research is needed to fully elucidate the etiology, it has been shown that abnormal placentation, immune dysregulation, and maternal inflammatory response appear to be involved. Genetic factors, environmental issues, and epigenetic modifications all play roles collaboratively according to recent studies (3).

Moreover, the burden of atopic diseases is increasing worldwide. The prevalence is on the rise in both industrialized and developing countries (4–7). Atopic diseases are reported to be associated with an imbalance between T helper 1 and 2. Increased T helper 2 can drive an inflammatory response. Immune reactions are thus exaggerated (8, 9).

The developmental origins of health and disease (DOHaD) hypothesis indicates a link between periconceptual, prenatal, early extrauterine environmental stress, and life-time health in offspring (10). DNA and hormones respond to early challenges, resulting in adaptations that can cause trans-generational developmental changes (11). Thus, the perinatal inflammation process caused by gestational hypertension may influence the phenotype expression in offspring. Gestational hypertension can lead to abnormal placentation, immune dysregulation, and maternal inflammatory responses during the prenatal and perinatal periods (12). In utero exposure to these stressors may elicit persistent consequences through epigenetic modifications, such as DNA methylation patterns (13). These epigenetic alterations are postulated to play a contributory role in the etiology of diseases that manifest in later stages of life (14). There are evidences supporting for a linkage between gestational hypertension and atopic diseases, primarily attributable to the downregulation of anti-inflammatory mediators and an augmentation of placental inflammation (12).

Currently, most original articles and meta-analyses focus on the association between asthma in offspring and complications of gestational hypertension, such as preeclampsia, which has been identified as an independent factor for asthma in childhood (15–17). However, the strength of the association between gestational hypertension and offspring atopic diseases remains elusive. Previous reported results are controversial (18–20). The aim of this study was to conduct a nationwide longitudinal follow-up investigation to elucidate the association between gestational hypertension and atopic diseases.

## Material and methods

### Database sources

The primary data were retrieved from Taiwan's National Health Insurance Research Database (NHIRD). NHIRD was established in 2002 and contains all claims data from Taiwan's National Health Insurance (NHI) system, a single-payer program with mandatory enrollment that was launched in 1995. The NHI has a coverage rate of more than 99% in Taiwan. It provides an invaluable resource for researchers studying a wide range of health issues in Taiwan (21).

Since 2015, the Health and Welfare Data Center (HWDC) of Taiwan's Ministry of Health and Welfare (MOHW) further merged the NHIRD and other health-related databases to strength data analyses. In this study, we analyzed the Maternal and Child Health Database (MCHD), which combines Taiwan's Birth Registration Database (BRD), the Birth Certificate Application (BCA) database, the National Register of Death (NRD), and the NHIRD. MCHD is a comprehensive health database that contains health and medical information for all Taiwanese women who have given birth, as well as their children. This database provides a comprehensive view of maternal and child health in Taiwan and can be used to generate accurate and reliable links between mothers and children (21–23).

Diagnoses in the NHIRD are coded using the International Classification of Diseases, both Ninth Revision Clinical Modification (ICD-9-CM) and Tenth Revision Clinical Modification (ICD-10-CM) format. Researchers are required to perform the analysis on site at HWDC through a remote connection to the MOHW server to protect patients’ identity and to verify databases. The study protocol was approved by the institutional review board of Taichung Veterans General Hospital (CE-17178A-5).

### Study population

**Figure 1:**
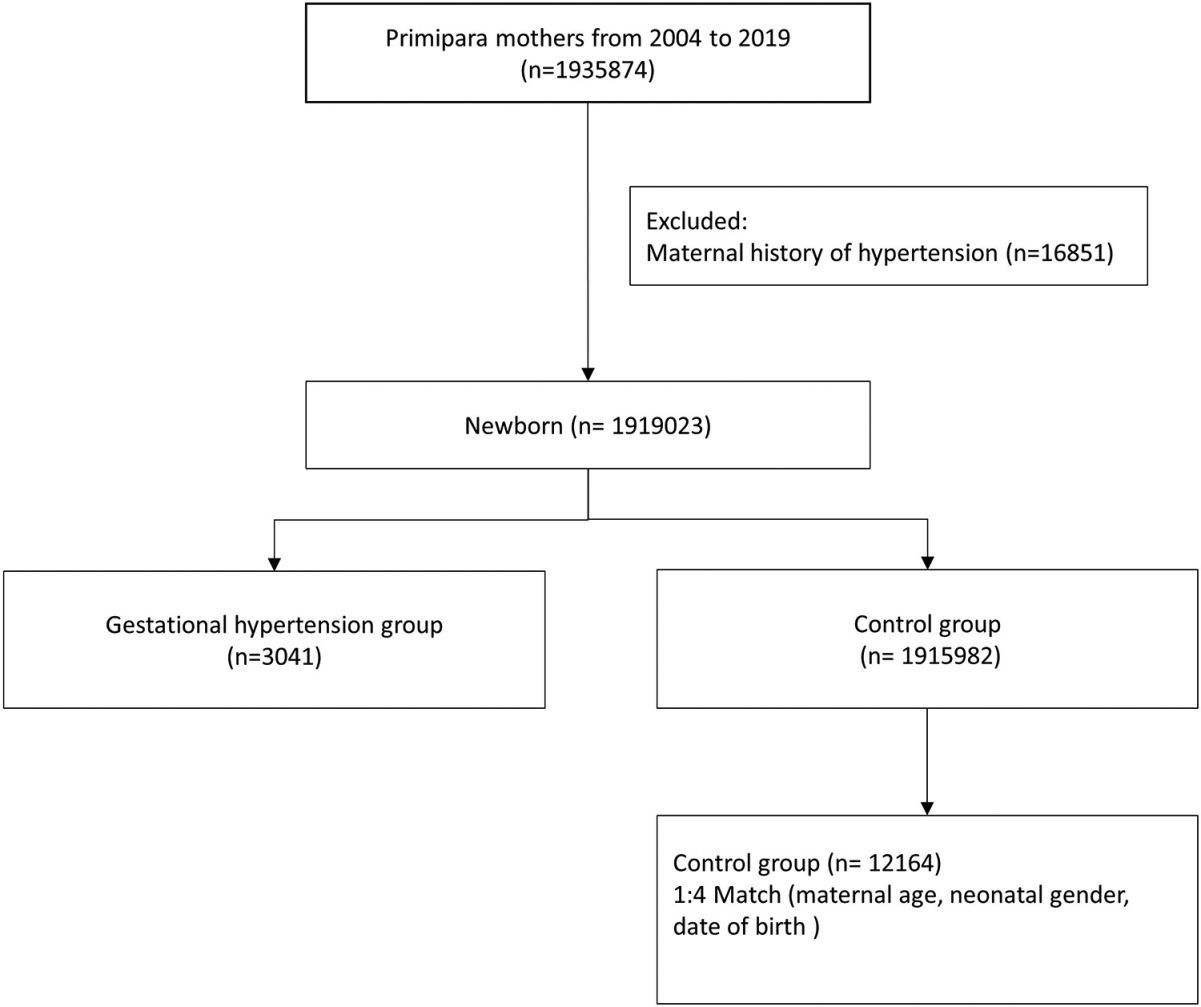
Composition of the study cohort.

This cohort included first-time mothers who were pregnant between 1 January 2004 and 31 December 2019. Mothers with diagnosis of gestational hypertension (ICD-9 code 642.3 and ICD-10 code O13, O16) were identified as cases, which excluded mothers with a history of chronic hypertension in their medical records. The control groups were matched to the cases by maternal age, neonatal gender, date of birth, and were randomly chosen at a control-to-case ratio of 4:1 (Figure 1).

### Outcome measurements

Each child was reviewed to confirm the diagnosis of atopic disease using ICD-9-CM code or ICD-10-CM code. Diseases described as atopic included asthma (ICD-9 code 493 and ICD-10 code J45), allergic rhinitis (ICD-9 code 477 and ICD-10 code J30), atopic dermatitis (ICD-9 code 691 and ICD-10 code L20), and urticaria (ICD-9 code 708 and ICD-10 code L50). The primary outcome was any atopic disorder documented in at least three outpatient visits or one admission.

### Covariates

Covariates included both maternal and neonatal factors. Maternal factors comprised maternal age, family income, mode of delivery, maternal comorbidity and pregnancy-related complications. Maternal comorbidities included hyperlipidemia, asthma, allergic rhinitis, atopic dermatitis, and urticaria. Pregnancy-related complications included preeclampsia, eclampsia, and gestational diabetes. The neonatal factors consisted of neonatal gender, number of babies, gestational age, and birth body weight.

### Statistical analysis

Data retrieval and analysis were conducted by SAS 9.4 (SAS Institute Inc). Student *t* tests were applied for continuous variables. Categorical variables are displayed as percentages and were compared using *χ*^2^ test. Using the Kaplan–Meier method, the cumulative survival incidence of each outcome was computed and visualized. The hazard ratio (HR) and 95% confidence intervals (CI) were estimated using the Cox proportional hazards regression model.

## Results

A total of 1,935,874 primiparas were enrolled in the study. After excluding 16,851 mothers with a history of hypertension, a total of 1,919,023 offspring were included in the study for the period 2004–2019. A total of 12,164 first-time mothers without gestational hypertension (1:4), matched by maternal age, neonatal gender, and date of birth, were randomly selected to serve as the control group. Demographic data of the control group and gestational hypertension group are shown in Table 1. The mothers in the gestational hypertension group had a similar distribution in urbanization and comorbidities, such as allergic rhinitis, atopic dermatitis, and urticaria. The group with gestational hypertension had higher rates of asthma, Cesarean section, gestational diabetes, hyperlipidemia, and twin birth rate. The family income in the gestational hypertension group was slightly lower. It is worth mentioning that this group also had a higher proportion of preeclampsia, preterm birth, and low birth weight infants.

**Table 1:** Demographic data of control group and gestational hypertension group.

Characteristic	Control Group	Gestational hypertension Group	Total	*P*-value
	(*n *= 12,164)	(*n* = 3,041)		
	*n* (%)	*n* (%)	*n* (%)	
Maternal Factors				
Maternal age				1.000
<25	972 (8)	243 (8)	1,215	
25–29	2,828 (23.2)	707 (23.2)	3,535	
30–34	4,624 (38.0)	1,156 (38)	5,780	
≥35	3,740 (30.7)	935 (30.7)	4,675	
Family income^a^				0.009
$≤18,780	2,293 (18.9)	554 (18.2)	2,847	
$18,781–27,600	4,403 (36.2)	1,182 (38.9)	5,585	
$27,601–42,000	2,979 (24.5)	752 (24.7)	3,731	
$>42,000	2,489 (20.5)	553 (18.2)	3,042	
Urbanization				0.12
Urban	7,803 (64.1)	1,905 (62.6)	9,708	
Suburban	1,436 (11.8)	349 (11.5)	1,785	
Rural	2,925 (24)	787 (25.9)	3,712	
Mode of delivery				<0.001
Vaginal delivery	7,639 (62.8)	1,179 (38.8)	8,818	
Cesarean section	4,525 (37.2)	1,862 (61.2)	6,387	
Maternal comorbidity				
Hyperlipidemia	262 (2.2)	161 (5.3)	423	<0.001
asthma	554 (4.6)	180 (5.9)	734	0.002
allergic rhinitis	3,931 (32.3)	1,004 (33)	4,935	0.46
atopic dermatitis	502 (4.1)	137 (4.5)	639	0.35
urticaria	2,808 (23.1)	707 (23.2)	3,515	0.85
Pregnancy-related complication				
Preeclampsia or eclampsia	45 (0.4)	254 (8.4)	299	<0.001
Gestational diabetes	229 (1.9)	255 (8.4)	484	<0.001
Neonatal Factors				
Neonatal Gender				1.000
Female	5,780 (47.5)	1,445 (47.5)	7,225	
Male	6,384 (52.5)	1,596 (52.5)	7,980	
Number of babies				<0.001
Singleton	11,859 (97.5)	2,895 (95.2)	14,754	
Multiple	305 (2.5)	146 (4.8)	451	
Gestational age				<0.001
>37 wks	11,097 (91.2)	2,271 (74.7)	13,368	
34–36 + 6 wks	963 (7.9)	640 (21)	1,603	
28–33 + 6 wks	58 (0.5)	85 (2.8)	143	
<28 wks	46 (0.4)	45 (1.5)	91	
Birth body weight				<0.001
>2,500 g	11,085 (91.1)	2,287 (75.2)	13,372	
1,500 g–2,500 g (LBW)	871 (7.2)	559 (18.4)	1,430	
<1,500 g (VLBW)	167 (1.4)	176 (5.8)	343	
<1,000 g (ELBW)	41 (0.3)	19 (0.6)	60	

^a^ Family income values by New Taiwan Dollars.

### Cumulative rates of atopic diseases

**Figure 2:**
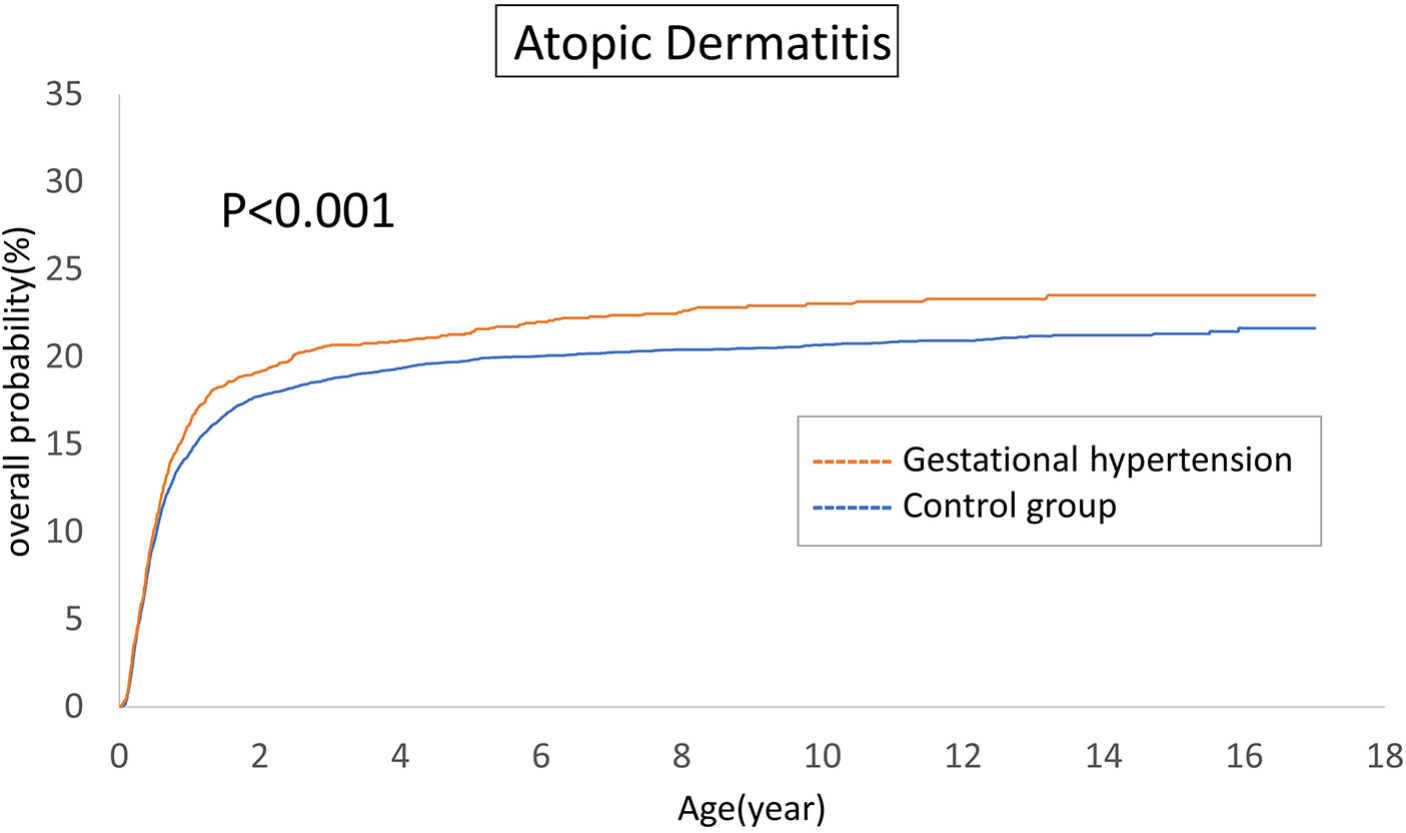
Comparison of the cumulative incidences of atopic dermatitis.

**Figure 3:**
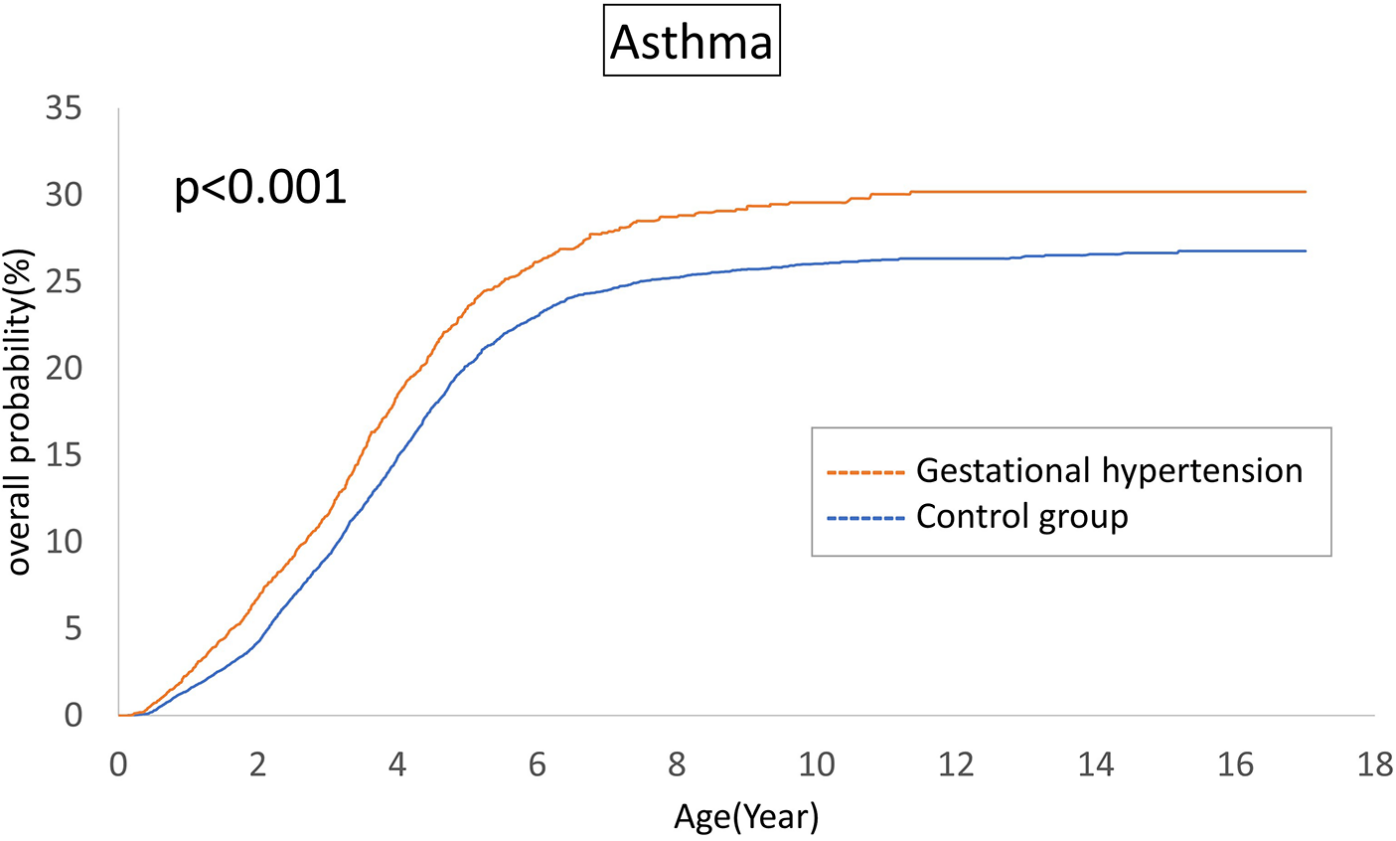
Comparison of the cumulative incidences of asthma.

**Figure 4:**
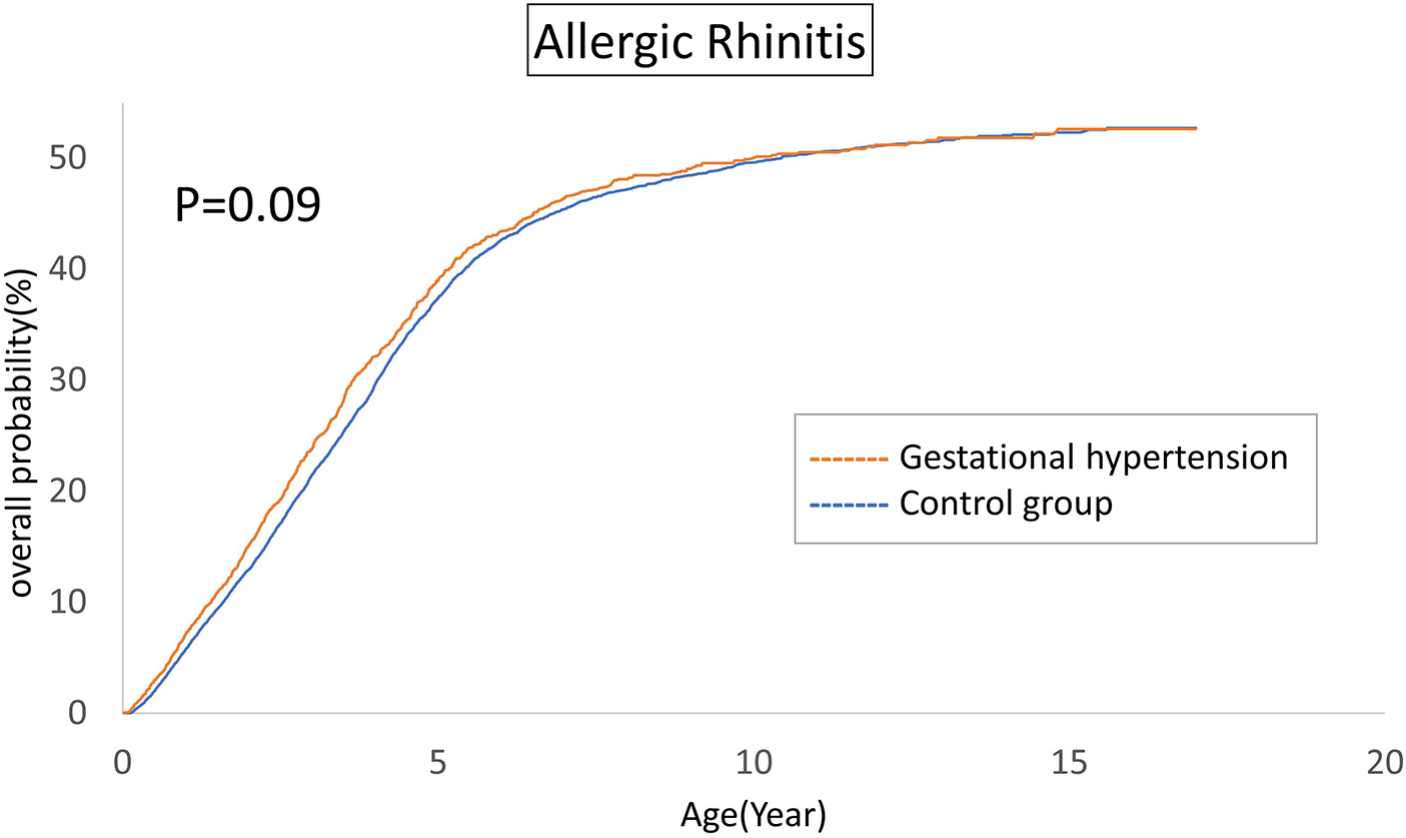
Comparison of the cumulative incidences of allergic rhinitis.

**Figure 5:**
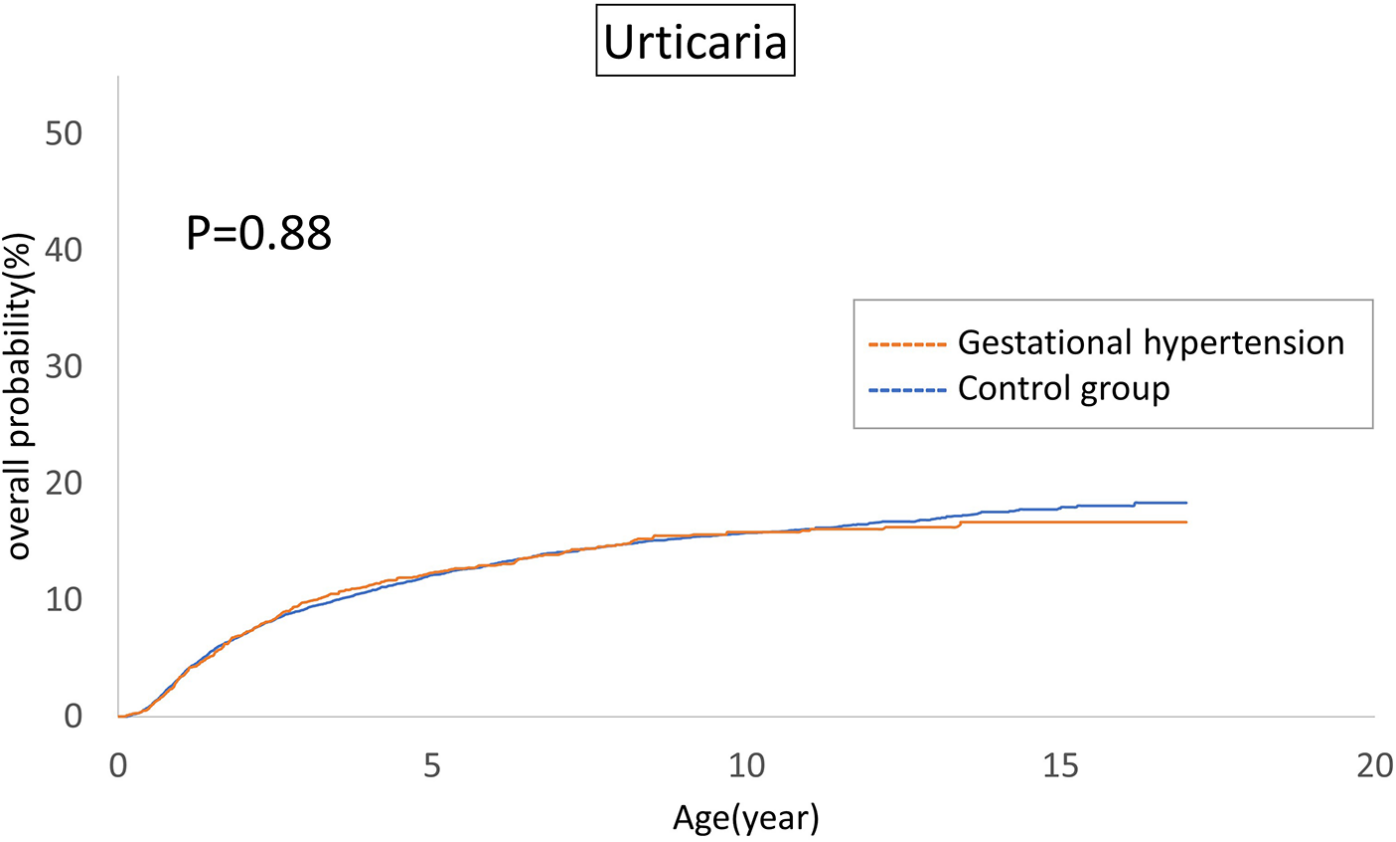
Comparison of the cumulative incidences of urticaria.

The cumulative incidences of atopic diseases were demonstrated from Figures 2 to 5. Offspring born to mothers afflicted with gestational hypertension exhibited a statistically significant higher risk of early-onset atopic conditions, specifically atopic dermatitis (as Figure 2) and asthma (Figure 3). Conversely, with regard to atopic diseases that manifest in later life, no statistically significant disparity was observed in the cumulative incidences of allergic rhinitis (Figure 4) and urticaria (Figure 5) between these two groups.

### Multiple model analysis for risk of atopic diseases

The results of the Cox regression model are summarized in Table 2. After adjusting for potential confounding factors, gestational hypertension still increased the risk of asthma (HR, 1.12, 95% CI, 1.02–1.23) and atopic dermatitis (HR, 1.10, 95% CI, 1.00–1.21). Nevertheless, gestational hypertension did not play an independent factor for allergic rhinitis (HR, 1.02, 95% CI, 0.95–1.10) and urticaria (HR, 1.02, 95% CI, 0.91–1.15). Regarding other factors, there were no differences among groups in terms of mother ages, family income, gestational ages, multiple births, preterm labor, and low birth body weight. Rural region was a protective factor for allergic rhinitis and atopic dermatitis. Cesarean resection was associated with increased risk of asthma and allergic rhinitis. Maternal asthma and allergic rhinitis contributed to asthma and allergic rhinitis in offspring. Maternal atopic dermatitis and urticaria were only associated with atopic dermatitis in offspring. Maternal urticaria increased risk of all atopic diseases in their children. Male gender was correlated with greater risk of atopic diseases, except for atopic dermatitis. Preeclampsia and eclampsia did not increase the risk of atopic diseases. Conversely, preeclampsia was protective for urticaria in offspring.

**Table 2:** Multivariable analysis of factors associated with atopic diseases.

Variables	Asthma			Allergic rhinitis			Atopic dermatitis			Urticaria		
	HR	95% CI	*P-value*	HR	95% CI	*P-value*	HR	95% CI	*P-value*	HR	95% CI	*P-value*
Gestational hypertension	1.12	1.02–1.23	0.019	1.02	0.95–1.10	0.55	1.10	1.00–1.21	0.041	1.02	0.91–1.15	0.73
Maternal age												
<25	1.00			1.00				1.00				1.00
25–29	1.04	0.89–1.20	0.64	1.23	1.10–1.38	0.000	1.12	0.96–1.30	0.14	0.95	0.80–1.13	0.56
30–34	0.96	0.83–1.11	0.60	1.22	1.09–1.36	0.000	1.13	0.98–1.31	0.10	0.98	0.83–1.16	0.83
≥35	0.90	0.78–1.05	0.17	1.15	1.03–1.29	0.016	0.96	0.83–1.12	0.61	0.87	0.73–1.04	0.14
Family income												
$≤18,780	1.00			1.00			1.00			1.00		
$18,781–27,600	1.05	0.95–1.16	0.37	0.97	0.90–1.04	0.42	0.86	0.78–0.95	0.004	0.92	0.81–1.04	0.17
$27,601–42,000	1.17	1.04–1.31	0.007	1.03	0.95–1.12	0.42	0.92	0.83–1.03	0.13	0.98	0.86–1.12	0.81
$>42,000	1.19	1.05–1.34	0.006	1.02	0.93–1.11	0.67	0.92	0.82–1.04	0.18	0.79	0.68–0.92	0.003
Urbanization												
Urban	1.00			1.00				1.00				1.00
Suburban	1.00	0.89–1.12	0.98	1.02	0.94–1.11	0.67	0.89	0.80–1.00	0.06	0.99	0.86–1.14	0.85
Rural	0.97	0.88–1.06	0.44	0.90	0.85–0.96	0.002	0.86	0.78–0.93	0.001	0.92	0.82–1.02	0.12
Mode of delivery												
Vaginal delivery	1.00			1.00			1.00			1.00		
Cesarean section	1.14	1.05–1.23	0.001	1.11	1.05–1.18	0.000	1.06	0.98–1.14	0.17	1.04	0.94–1.14	0.48
Maternal comorbidity												
Hyperlipidemia	1.16	0.94–1.44	0.16	0.91	0.76–1.07	0.25	1.08	0.88–1.33	0.47	0.98	0.74–1.30	0.89
asthma	1.27	1.08–1.49	0.003	1.18	1.04–1.33	0.010	1.11	0.95–1.31	0.20	1.06	0.86–1.31	0.60
allergic rhinitis	1.27	1.17–1.38	<0.001	1.34	1.26–1.42	<0.001	1.01	0.93–1.09	0.85	1.00	0.91–1.11	0.99
atopic dermatitis	0.99	0.82–1.20	0.93	1.03	0.90–1.18	0.65	1.41	1.21–1.65	<0.001	0.92	0.73–1.16	0.48
urticaria	1.13	1.04–1.23	0.006	1.08	1.02–1.16	0.013	1.20	1.11–1.30	<0.001	1.44	1.30–1.59	<0.001
Pregnancy-related complication												
Preeclampsia or eclampsia	0.85	0.65–1.12	0.25	1.11	0.92–1.35	0.28	1.05	0.81–1.35	0.74	0.65	0.43–0.98	0.037
Gestational diabetes	0.97	0.79–1.20	0.79	0.91	0.78–1.07	0.26	1.02	0.83–1.25	0.86	0.92	0.70–1.21	0.55
Neonatal Gender												
Female	1.00			1.00			1.00			1.00		
Male	1.33	1.23–1.43	<0.001	1.28	1.21–1.35	<0.001	1.07	0.99–1.15	0.08	1.11	1.02–1.22	0.02
Number of babies												
Singleton	1.00			1.00			1.00			1.00		
Multiple	0.81	0.64–1.03	0.080	0.95	0.80–1.12	0.53	0.97	0.77–1.22	0.78	0.65	0.47–0.92	0.014
Gestational age												
>37 wks	1.00			1.00			1.00			1.00		
34–36 + 6 wks	1.03	0.89–1.19	0.69	0.97	0.88–1.08	0.61	0.85	0.73–0.98	0.03	0.96	0.80–1.15	0.62
28–33 + 6 wks	1.04	0.69–1.56	0.87	0.98	0.70–1.36	0.89	1.00	0.64–1.55	1.00	0.57	0.32–1.02	0.06
<28 wks	1.50	0.79–2.86	0.21	1.03	0.59–1.79	0.93	0.82	0.37–1.80	0.62	0.29	0.08–1.03	0.06
Birth body weight												
>2,500 g	1.00			1.00			1.00			1.00		
1,500 g–2,500 g (LBW)	1.22	1.06–1.39	0.005	1.06	0.96–1.18	0.27	1.09	0.95–1.26	0.22	1.05	0.88–1.26	0.57
<1,500 g (VLBW)	1.30	0.97–1.75	0.09	1.12	0.89–1.42	0.33	1.06	0.76–1.47	0.74	1.79	1.26–2.55	0.001
<1,000 g (ELBW)	1.78	0.78–4.08	0.17	1.10	0.52–2.32	0.81	0.68	0.22–2.08	0.50	1.51	0.26–8.99	0.65

## Discussion

In this population-based longitudinal follow-up study, we found maternal hypertension increased risk of asthma and atopic dermatitis in offspring. Preeclampsia and gestational diabetes were not significantly associated with atopic disease. The major strengths of this study are the large number of case numbers and the comprehensive longitudinal follow-up for 16 years. Furthermore, a number of variables pertaining to baby-mother relations, including maternal age, socioeconomic status, urbanization, mode of delivery, maternal comorbidities, complications of pregnancy, neonatal gender, numbers of babies, and preterm labor were also considered as potential confounding factors when analyzing the data.

Henderson et al. conducted a cohort study in the United Kingdom, and found that pregnancy-induced hypertension was weakly associated with asthma and contributed to preterm labor. There was a weak association between gestational hypertension and asthma after additionally adjusting for potential cofounders, cesarean delivery and gestational age. However, long-term outcomes were not documented in this study (20).

In a study conducted in Norway, Byberg et al. reported that the offspring of mothers who experienced complication of gestational hypertension had an increased risk of allergic sensitization and atopic rhino-conjunctivitis, but this was not associated with asthma and atopic dermatitis. This cohort collected 617 children who were 1:2 matched between 1993 and 1995, and then followed up 10–12 years later. The follow-up included two types of measurement. The first follow-up was conducted using a questionnaire to determine whether the children had ever been diagnosed with an atopic disease, such as allergic rhino-conjunctivitis, atopic dermatitis, or asthma. The second follow-up included laboratory measurements, i.e., allergic sensitization and lung function test. The present study had a longer follow-up period, but was restricted to the effects of severe gestational hypertension with complications. The other limitation was that the definition of atopic disease was done by questionnaire only (24).

The developmental origins of health and disease (DOHaD) indicated a link between *in utero* environmental stress and life-time health, including vulnerability to disease in offspring (10). The concept emerged from research conducted on a population of 300,000 men in the Netherlands, who were born to a group of mothers during a period of food shortage in the 1930 s. The intrauterine malnutrition contributed to different disease patterns, metabolic health issues, and even mental problems. Recently, Msallam et al. (2020) conducted a study on mice and demonstrated that fetal mast cell are reactivated in the airway after exposure to maternal IgE-mediated sensitization *in utero* by crossing the placental barrier (25, 26).

Atopic diseases are common among children, adolescents, and adults in modern society. It represents a group of diseases associated with an uncoordinated immune system, including asthma, atopic rhinitis, atopic dermatitis (eczema), and urticaria (most related to food allergy). In Taiwan, the prevalence rates of asthma, atopic rhinitis, eczema, and urticaria are 11.9%, 26.3%, 6.7%, and 0.8%, respectively (4, 27). The theory of atopic march includes genetic factors and environmental factors. No single factor can be solely attributed to the cause. From a genetic perspective, atopic disease is a heterogeneous disease. It could also be modified by epigenetic factors, including DNA methylation, histone acetylation, and certain specific non-coding RNAs. Epigenetic regulation of disease can also be influenced by environmental factors (28). The intrauterine environment is known to play a role in this regard. Perinatal factors, including maternal age, maternal comorbidities, complications of pregnancy, and even maternal nutrition, influence the intrauterine environment, which contributes to the development of health disorders of the offspring in later life, such as atopic disease, hypertension, diabetes, mood disorders, and other chronic health problems (29).

Hypertensive disorder in pregnancy (HDP) occurs in up to 5%–10% of pregnancies, with 35% of HDP cases developing complications, such as preeclampsia or eclampsia (1). Women have a lower tolerance to high blood pressure during pregnancy, and thus hypertensive encephalopathy could happen at a lower blood pressure. According to the American College of Obstetricians and Gynecologists (ACOG), “Severe hypertension is defined as systolic blood pressure (SBP) ⩾160 mmHg and/or diastolic blood pressure (DBP) ⩾110 mmHg.” This is clearly stricter than the guideline for the general population, which defines severe hypertension as SBP ⩾ 180 mmHg and/or DBP ⩾ 110 mmHg. The pathophysiology has not been comprehensively established, but it is generally believed that placenta dysregulation brings about systemic vascular endothelial dysfunction (3, 12). The angiogenic imbalance causes vascular endothelial growth factor (VEGF) and placental growth factor (PIGF) to increase. VEGF and PIGF both reduce nitric oxide synthesis and contribute to placenta ischemia by vascular remodeling and vasodilation (30). Placental ischemia also makes CD4+ T cells increase and T regulatory cells decrease, which leads to immune imbalance in the mother and in the intrauterine environment (31). This imbalance is associated with chronic inflammation, which may affect the development of the immune system of the fetus as evidenced by the presence of maternal immune cells and increased cytokines level, such as interleukin-4 (IL4), interleukin-6 (IL6), and interleukin-10 (IL10) in neonates (32, 33). From an immunological standpoint, gestational hypertension can be interpreted as a manifestation of a rejection response. This is evidenced by the suppression of T helper 2 cells, known for their capacity to inhibit natural killer (NK) cell and T helper 1 cell activation, in mothers afflicted by gestational hypertension. Moreover, this response is potentiated by the antibody-mediated immune response orchestrated by B cells (34, 35). These profound perturbations within the fetal immune system may exert enduring epigenetic influences on individuals' health, potentially culminating in the onset of atopic diseases and other protracted health repercussions.

The atopic march in infancy and childhood resembles a mixture of exogenous and hereditary factors. The atopic march proposes that atopic dermatitis generated in infancy and may progress to asthma and allergic rhinitis in specific individuals. The pathways of atopic march involve imbalances in Th1/Th2 immune responses, IgE-mediated and non-IgE-mediated immune reactions (36). In our investigation, we indeed identified a correlation between gestational hypertension and an elevated cumulative incidence of atopic dermatitis and asthma. However, we could not establish a significant association between allergic rhinitis and urticaria within the context of the atopic march. This observation may indicate that the influence of gestational hypertension is predominantly concentrated in the early stages of life. For atopic conditions that manifest later in life, it is plausible that environmental and genetic factors assume more prominent roles.

This study has certain limitations. First, the diagnoses were dependent on the physicians' coding. The validity of the diagnoses could not be verified due to privacy protocols. Furthermore, laboratory data were not included in the claims data. Hence, there might have been misclassifications. Second, the severity of gestational hypertension, gestational diabetes, and preeclampsia could not be clearly identified using data from the NHIRD. Various degrees of illness might have resulted in varying levels of inflammatory response and cytokine release.

## Conclusion

Maternal gestational hypertension increased the cumulative risk of asthma and atopic dermatitis in offspring. However, there was no increased risk of atopic rhinitis and urticaria. Additional prospective studies are essential to provide clarity on the correlation and the underlying epigenetic mechanisms relevant to this issue.

## Data Availability

The data analyzed in this study is subject to the following licenses/restrictions: To protect patients’ identity and validate the reliability of the databases, investigators are required to perform onsite analysis at HWDC via remote connection to MOHW servers. Requests to access these datasets should be directed to Ching-Heng Lin, epid@vghtc.gov.tw.

## References

[1] The American College of Obstetricians and Gynecologists (ACOG). Gestational hypertension and preeclampsia: ACOG practice bulletin summary, number 222. Obstet Gynecol. (2020) 135(6):1492–5. 10.1097/aog.0000000000003892 32443077

[2] Rana S Lemoine E Granger JP Karumanchi SA. Preeclampsia: pathophysiology, challenges, and perspectives. Circ Res. (2019) 124(7):1094–112. 10.1161/circresaha.118.313276 30920918

[3] Braunthal S Brateanu A. Hypertension in pregnancy: pathophysiology and treatment. SAGE Open Med. (2019) 7:2050312119843700. 10.1177/2050312119843700 31007914 PMC6458675

[4] Kuo HC Chu CH Su YJ Lee CH. Atopic dermatitis in Taiwanese children: the laboratory values that correlate best to the SCORAD index are total IgE and positive cheddar cheese IgE. Medicine. (2020) 99(30):e21255. 10.1097/md.0000000000021255 32791702 PMC7387036

[5] Lai CK Beasley R Crane J Foliaki S Shah J Weiland S. Global variation in the prevalence and severity of asthma symptoms: phase three of the international study of asthma and allergies in childhood (ISAAC). Thorax. (2009) 64(6):476–83. 10.1136/thx.2008.106609 19237391

[6] Urban K Chu S Giesey RL Mehrmal S Uppal P Nedley N The global, regional, and national burden of atopic dermatitis in 195 countries and territories: an ecological study from the global burden of disease study 2017. JAAD Int. (2021) 2:12–8. 10.1016/j.jdin.2020.10.002 34409347 PMC8362298

[7] Loh W Tang MLK. The epidemiology of food allergy in the global context. Int J Environ Res Public Health. (2018) 15(9):2043. 10.3390/ijerph15092043 30231558 PMC6163515

[8] Georas SN Guo J De Fanis U Casolaro V. T-helper cell type-2 regulation in allergic disease. Eur Respir J. (2005) 26(6):1119–37. 10.1183/09031936.05.00006005 16319345

[9] Akdis M Trautmann A Klunker S Daigle I Kucuksezer UC Deglmann W T helper (Th) 2 predominance in atopic diseases is due to preferential apoptosis of circulating memory/effector Th1 cells. Faseb J. (2003) 17(9):1026–35. 10.1096/fj.02-1070com 12773485

[10] Silveira PP Portella AK Goldani MZ Barbieri MA. Developmental origins of health and disease (DOHaD). J Pediatr. (2007) 83(6):494–504. 10.2223/jped.1728 18074050

[11] Aiken CE Ozanne SE. Transgenerational developmental programming. Hum Reprod Update. (2014) 20(1):63–75. 10.1093/humupd/dmt043 24082037

[12] Harmon AC Cornelius DC Amaral LM Faulkner JL Cunningham MW Wallace K The role of inflammation in the pathology of preeclampsia. Clin Sci. (2016) 130(6):409–19. 10.1042/cs20150702 PMC5484393 26846579

[13] De Borre M Che H Yu Q Lannoo L De Ridder K Vancoillie L Cell-free DNA methylome analysis for early preeclampsia prediction. Nat Med. (2023) 29(9):2206–15. 10.1038/s41591-023-02510-5 37640858

[14] Yang C Baker PN Granger JP Davidge ST Tong C. Long-term impacts of preeclampsia on the cardiovascular system of mother and offspring. Hypertension. (2023) 80(9):1821–33. 10.1161/hypertensionaha.123.21061 37377011

[15] Conlan N Maher GM Al Khalaf SY McCarthy FP Khashan AS. Association between hypertensive disorders of pregnancy and the risk of asthma, eczema and allergies in offspring: a systematic review and meta-analysis. Clin Exp Allergy. (2021) 51(1):29–38. 10.1111/cea.13754 33037716

[16] Stokholm J Sevelsted A Anderson UD Bisgaard H. Preeclampsia associates with asthma, allergy, and eczema in childhood. Am J Respir Crit Care Med. (2017) 195(5):614–21. 10.1164/rccm.201604-0806OC 27626972

[17] Nahum Sacks K Friger M Shoham-Vardi I Sergienko R Landau D Sheiner E. In utero exposure to pre-eclampsia as an independent risk factor for long-term respiratory disease. Pediatr Pulmonol. (2020) 55(3):723–8. 10.1002/ppul.24659 31985889

[18] Algert CS Bowen JR Lain SL Allen HD Vivian-Taylor JM Roberts CL. Pregnancy exposures and risk of childhood asthma admission in a population birth cohort. Pediatr Allergy Immunol. (2011) 22(8):836–42. 10.1111/j.1399-3038.2011.01206.x 21929593 PMC3263424

[19] Shaheen SO Macdonald-Wallis C Lawlor DA Henderson AJ. Hypertensive disorders of pregnancy, respiratory outcomes and atopy in childhood. Eur Respir J. (2016) 47(1):156–65. 10.1183/13993003.00643-2015 26541530 PMC4884643

[20] Henderson I Quenby S. Gestational hypertension and childhood atopy: a millennium cohort study analysis. Eur J Pediatr. (2021) 180(8):2419–27. 10.1007/s00431-021-04012-3 33770273 PMC8285347

[21] Hsieh CY Su CC Shao SC Sung SF Lin SJ Kao Yang YH Taiwan’s national health insurance research database: past and future. Clin Epidemiol. (2019) 11:349–58. 10.2147/clep.S196293 31118821 PMC6509937

[22] Lin MC Lai MS. Pediatricians’ role in caring for preschool children in Taiwan under the national health insurance program. J Formos Med Assoc. (2009) 108(11):849–55. 10.1016/s0929-6646(09)60416-2 19933028

[23] Wu TP Liang FW Huang YL Chen LH Lu TH. Maternal mortality in Taiwan: a nationwide data linkage study. PLoS One. (2015) 10(8):e0132547. 10.1371/journal.pone.0132547 26237411 PMC4523206

[24] Byberg KK Ogland B Eide GE Oymar K. Birth after preeclamptic pregnancies: association with allergic sensitization and allergic rhinoconjunctivitis in late childhood; a historically matched cohort study. BMC Pediatr. (2014) 14:101. 10.1186/1471-2431-14-101 24725676 PMC3995723

[25] Mass E Gentek R. Fetal-derived immune cells at the roots of lifelong pathophysiology. Front Cell Dev Biol. (2021) 9:648313. 10.3389/fcell.2021.648313 33708774 PMC7940384

[26] Msallam R Balla J Rathore APS Kared H Malleret B Saron WAA Fetal mast cells mediate postnatal allergic responses dependent on maternal IgE. Science. (2020) 370(6519):941–50. 10.1126/science.aba0864 33122426

[27] Chu CY Cho YT Jiang JH Lin EI Tang CH. Epidemiology and comorbidities of patients with chronic urticaria in Taiwan: a nationwide population-based study. J Dermatol Sci. (2017) 88(2):192–8. 10.1016/j.jdermsci.2017.07.006 28743610

[28] Nedoszytko B Reszka E Gutowska-Owsiak D Trzeciak M Lange M Jarczak J Genetic and epigenetic aspects of atopic dermatitis. Int J Mol Sci. (2020) 21(18):6484. 10.3390/ijms21186484 32899887 PMC7554821

[29] Peral-Sanchez I Hojeij B Ojeda DA Steegers-Theunissen RPM Willaime-Morawek S. Epigenetics in the uterine environment: how maternal diet and ART may influence the epigenome in the offspring with long-term health consequences. Genes. (2021) 13(1):31. 10.3390/genes13010031 35052371 PMC8774448

[30] Rana S Burke SD Karumanchi SA. Imbalances in circulating angiogenic factors in the pathophysiology of preeclampsia and related disorders. Am J Obstet Gynecol. (2022) 226(2s):S1019–34. 10.1016/j.ajog.2020.10.022 33096092 PMC8884164

[31] LaMarca B Amaral LM Harmon AC Cornelius DC Faulkner JL Cunningham MW Jr. Placental ischemia and resultant phenotype in animal models of preeclampsia. Curr Hypertens Rep. (2016) 18(5):38. 10.1007/s11906-016-0633-x 27076345 PMC5127437

[32] Smith SE Li J Garbett K Mirnics K Patterson PH. Maternal immune activation alters fetal brain development through interleukin-6. J Neurosci. (2007) 27(40):10695–702. 10.1523/jneurosci.2178-07.2007 17913903 PMC2387067

[33] Mor G Cardenas I. The immune system in pregnancy: a unique complexity. Am J Reprod Immunol. (2010) 63(6):425–33. 10.1111/j.1600-0897.2010.00836.x 20367629 PMC3025805

[34] Saito S Sakai M Sasaki Y Tanebe K Tsuda H Michimata T. Quantitative analysis of peripheral blood Th0, Th1, Th2 and the Th1: Th2 cell ratio during normal human pregnancy and preeclampsia. Clin Exp Immunol. (1999) 117(3):550–5. 10.1046/j.1365-2249.1999.00997.x 10469061 PMC1905376

[35] Zhou Q Wu Y Zhang D. Exploring the role of T helper subgroups and their cytokines in the development of pregnancy-induced hypertension. Front Immunol. (2023) 14:1126784. 10.3389/fimmu.2023.1126784 37342348 PMC10277627

[36] Gabryszewski SJ Hill DA. One march, many paths: insights into allergic march trajectories. Ann Allergy Asthma Immunol. (2021) 127(3):293–300. 10.1016/j.anai.2021.04.036 33971364 PMC8418995

